# Falciform ligament abscess with disseminated intrahepatic foci: a case report

**DOI:** 10.1186/s40792-022-01466-x

**Published:** 2022-06-14

**Authors:** Tadao Kuribara, Itaru Shigeyoshi, Tatsuo Ichikawa, Kiyoshi Osa, Takeshi Inoue, Satoshi Ono, Kozo Asanuma, Shiori Kaneko, Takayuki Sano, Kouta Matsubara, Naoko Irie, Kanako Suzuki, Akira Iai, Hideki Ishizu

**Affiliations:** 1 Department of Surgery, Saitama Cooperative Hospital, 1317 Kizoro, Kawaguchi-shi, Saitama, 333-0831 Japan; 2 Department of Pathology, Saitama Cooperative Hospital, 1317 Kizoro, Kawaguchi-shi, Saitama, Japan

**Keywords:** Falciform ligament, Round ligament, Abscess, Dissemination, Liver

## Abstract

**Background:**

Falciform ligament abscess (FLA) is a rare disease, and its diagnosis can be challenging without typical image findings of an abscess. We report a patient with FLA that presented as a mass, with an indistinct border between it and the liver, in addition to disseminated foci within the liver. This made it difficult to determine whether it was FLA or a malignancy.

**Case presentation:**

A 69-year-old man presented with epigastric pain. Contrast-enhanced computed tomography revealed a 25-mm mass below the middle of the diaphragm. Based on an initial diagnosis of infection of the falciform ligament, we administered conservative antibiotic treatment and there was initial improvement in the patient’s clinical condition and laboratory data. However, he continued to experience mild epigastric pain. A month later, imaging studies revealed enlargement of the falciform ligament mass and the emergence of a new nodule in the liver, whereas laboratory findings showed re-elevated C-reactive protein levels. Since conservative treatment had failed, we decided to perform surgery. Considering the imaging study findings, malignant disease could not be ruled out. Based on the operative findings, we performed combined resection of the falciform ligament, left liver, and gallbladder. Histopathological examination of the resected specimens revealed extensive neutrophil infiltration and the presence of giant cells and foam cells within the lesions. These findings were indicative of abscess. *Pseudomonas aeruginosa* was cultured from the pus in the falciform ligament mass and bile in the gallbladder. Although multiple abscesses postoperatively developed in the residual portion of the liver, they could be treated through antibiotic therapy.

**Conclusions:**

FLA can spread to both adjacent and distant organs via its rich vascular and lymphatic networks. When FLA displays atypical image findings and/or an atypical clinical course, it can be difficult to distinguish it from malignant disease. In such cases, surgical treatment, with intraoperative pathological diagnosis, should be attempted.

## Background

The falciform ligament is strongly connected to surrounding organs via rich vascular and lymphatic networks. Falciform ligament abscess (FLA) is a rare disease, typically caused by the transmission of bacteria from other organs via these networks. Diagnosis is relatively simple in cases with typical image findings, because an abscess usually presents on computed tomography (CT) scans or magnetic resonance imaging (MRI) as a mass with well-defined enhanced capsule wall and with internal fluid and air content. However, when an abscess has minimal fluid, it can be difficult to distinguish from a malignant tumor.

Since FLA results from bacteria transmitted to the falciform ligament from surrounding organs, it could also spread bacteria to surrounding or distant organs via the same vascular or lymphatic pathways. However, instances of this have not been reported.

Here, we report a patient with FLA that presented as a mass with disseminated foci in the liver and a border indistinct from the liver. These characteristics made the FLA difficult to differentiate from malignancy. This is the first report of FLA with disseminated intrahepatic foci.

## Case presentation

**Fig. 1:**
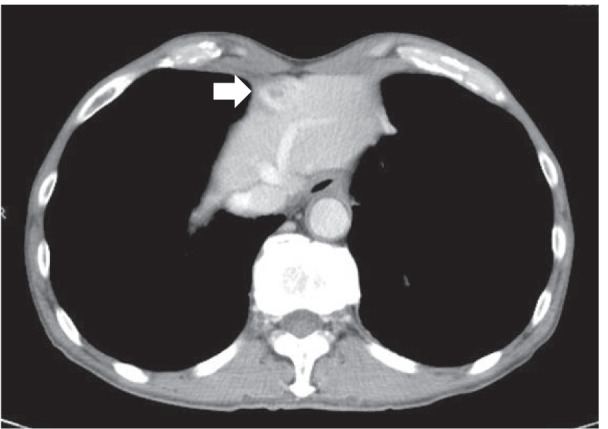
Contrast-enhanced computed tomography of the falciform ligament abscess at the time of the first admission. A 25-mm, enhanced mass containing small low-density areas was observed below the center of the diaphragm (white arrow)

**Fig. 2:**
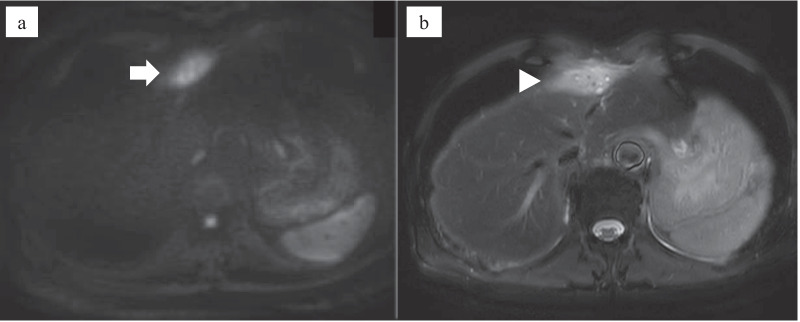
Magnetic resonance imaging (MRI) of the falciform ligament abscess at the time of the first admission. **a** The falciform ligament mass showed high signal intensity on diffusion-weighted images (white arrow). **b** Small high-intensity areas within the mass were visible on T2-weighted images (white arrowhead)

A 69-year-old man with no significant medical history presented to the emergency department with epigastric pain that had lasted for 5 days. On physical examination, he had a slight fever and mild tenderness around the epigastrium. Laboratory findings were unremarkable, except for mildly elevated C-reactive protein (CRP) levels of 6.9 mg/dl. Contrast-enhanced CT revealed a 25-mm enhanced mass with small low-density areas below the middle of the diaphragm (Fig. 1). On MRI, the signal intensity of the mass was high in diffusion-weighted images, and small high-intensity areas inside the mass were seen on T2-weighted images (Fig. 2).

**Fig. 3:**
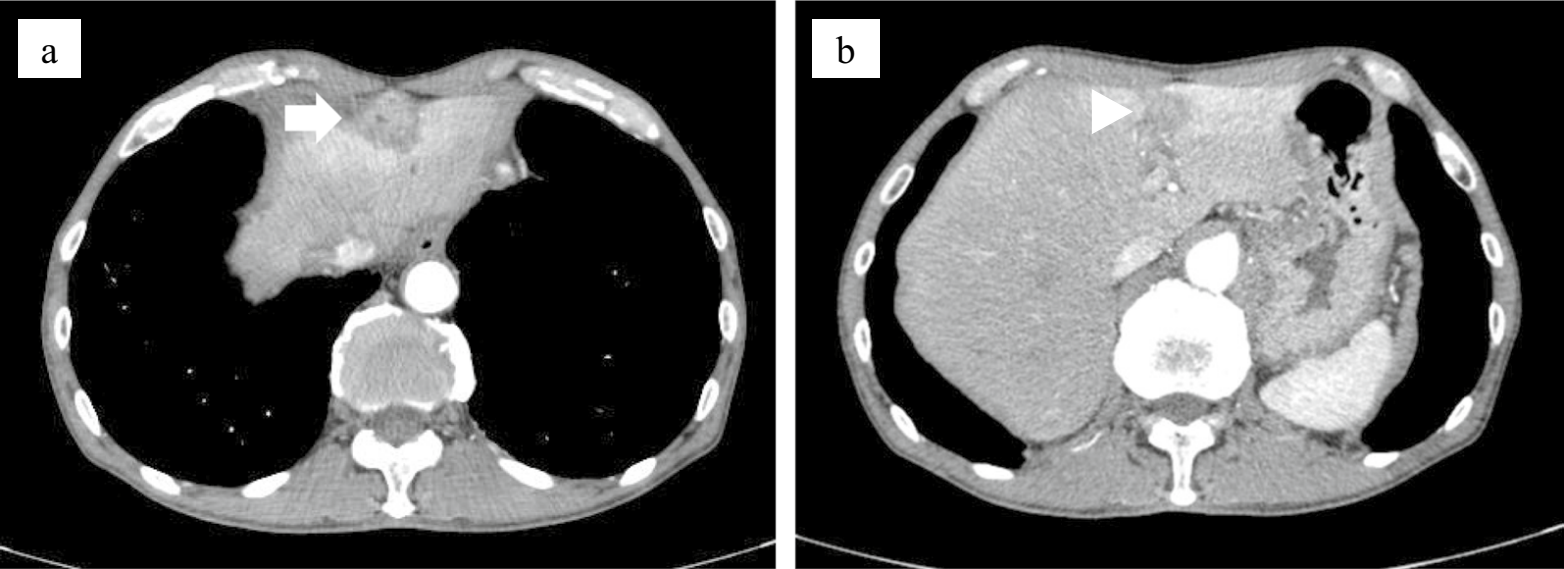
Computed tomography a month following the first discharge. **a** The falciform ligament mass (white arrow) had increased in size, and the border between the mass and the liver was obscure. **b** A low-density nodule had emerged in liver segment three (white arrowhead)

**Fig. 4:**
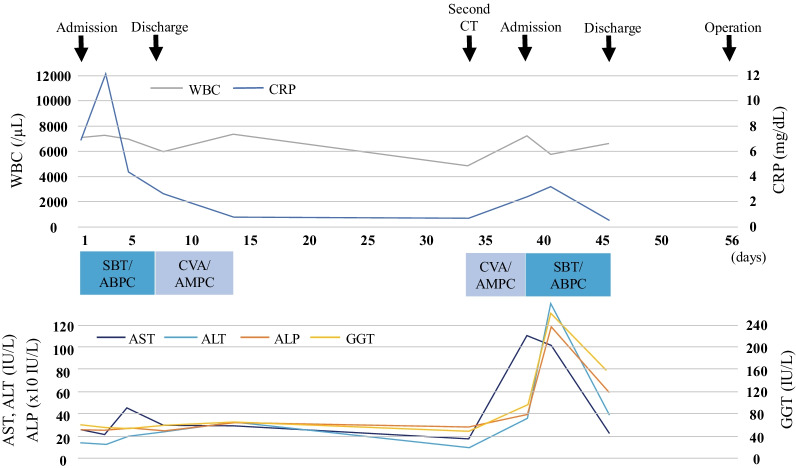
The clinical course of the patient after the first admission till the operation. *ALT* alanine aminotransferase, *ALP* alkaline phosphatase, *AST* aspartate aminotransferase, *CRP* C-reactive protein, *CT* computed tomography, *CVA/AMPC* clavulanic acid/amoxicillin, *GGT* gamma-glutamyltransferase, *SBT/ABPC* sulbactam/ampicillin, *WBC* white blood cell

Based on a diagnosis of falciform ligament infection, we administered conservative therapy in the form of sulbactam/ampicillin (SBT/ABPC) infusion and subsequent oral clavulanic acid/amoxicillin (CVA/AMPC). Performed at admission, the blood culture was negative. Imaging studies showed no apparent pancreatobiliary or other organ infections. The patient also had no biliary history. Additionally, the upper and lower gastrointestinal endoscopies revealed no significant findings. Therefore, the cause of FLA was unclear. The patient’s general condition and laboratory data improved, and he was discharged. However, he continued to complain of mild epigastric pain. A further CT scan was performed a month after discharge, and this revealed that the falciform ligament mass had increased in size. We also observed an obscure border between the mass and the liver and a new low-density nodule in liver segment three, with upstream bile duct dilatation (Fig. 3). After second CT, the patient was readmitted due to increased abdominal pain and re-elevation of his CRP levels. Since conservative treatment had failed, we decided to perform surgery. Although tests for tumor markers were negative, considering the changes to imaging study findings, malignant disease could not be ruled out. Figure 4 shows the clinical course of the patient after the first admission till the operation.

**Fig. 5:**
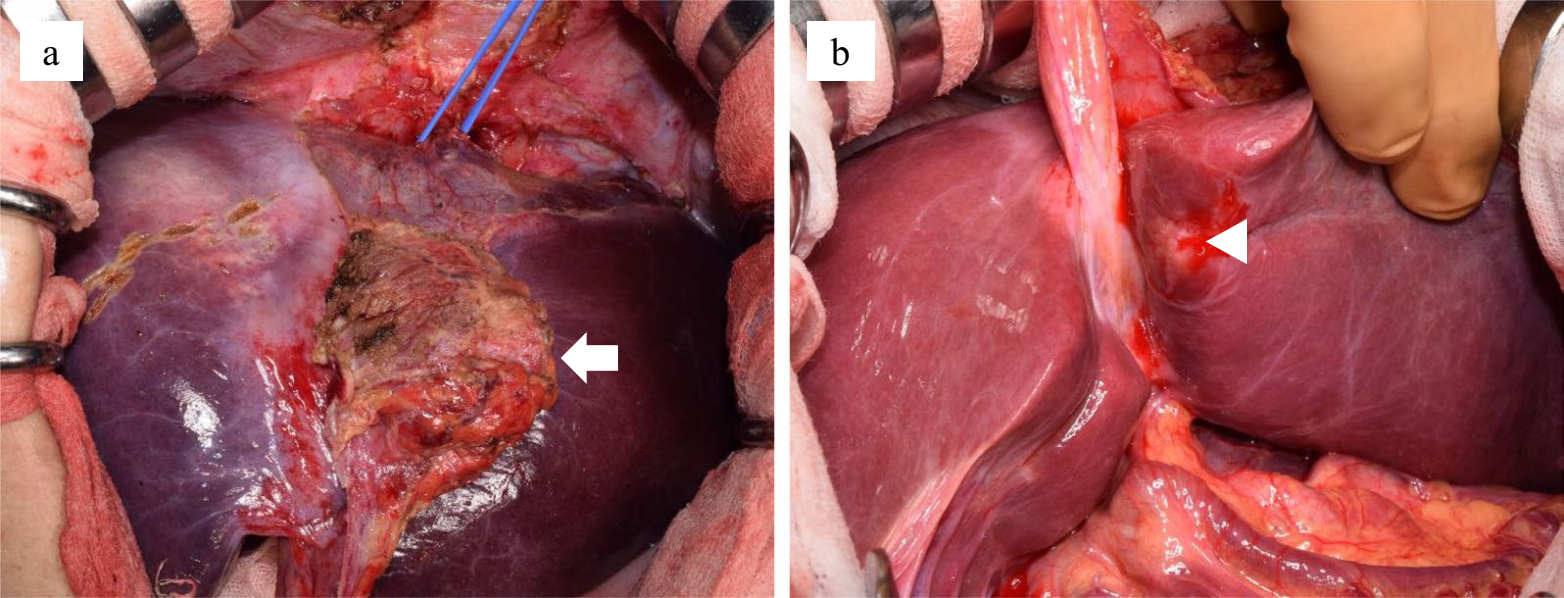
Operative findings. **a** The falciform ligament mass (white arrow). **b** A nodule emerging in liver segment three (white arrowhead)

During surgery, laparotomy and intraoperative ultrasonography confirmed our imaging findings, revealing an indistinct border between the mass of the falciform ligament and the liver, and a yellowish-white nodule in liver segment three (Fig. 5). We found no adhesion around the gallbladder and no gallbladder wall thickening. Additionally, the bile juice in the gallbladder, collected intraoperatively, was normal in color and odorless. Based on these findings, considering the possibility of malignancy, we decided to perform left hepatectomy and cholecystectomy.

**Fig. 6:**
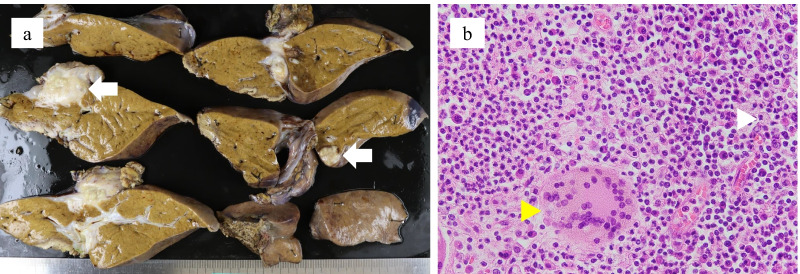
Histopathological examination of the resected specimens. **a** Gross findings showed the falciform ligament mass and the yellowish-white nodule in liver segment three (white arrows). **b** Microscopic examination revealed numerous neutrophils, giant cells (yellow arrowhead), and foam cells (white arrowhead)

The cut surfaces of the resected specimens showed both the falciform ligament mass and the liver segment three nodule to be pale white, irregularly shaped nodular lesions. Histopathological examination revealed extensive neutrophil infiltration and the presence of giant cells and foam cells in both lesions, consistent with abscesses (Fig. 6). *Pseudomonas aeruginosa* was cultured from the pus in the falciform ligament mass and the bile in the gallbladder. Although multiple abscesses developed postoperatively in the remaining liver, they rapidly improved with the use of antibiotics (levofloxacin) and the patient was discharged. He did not experience subsequent recurrence of intra-abdominal abscesses.

## Discussion

FLA is a rare condition, with only about 20 adult cases reported in the literature so far, including reports of ligamentum teres abscess. Table 1 shows the results of a Pub-Med database search using the terms “falciform ligament”, “ligamentum teres”, “round ligament”, “abscess”, and “gangrene”. FLA is typically caused by bacterial transmission from infected surrounding organs to the falciform ligament via their rich vascular or lymphatic networks. Although FLA would also be expected to spread infection to other organs through these networks in the same manner, there have been no reports of this in the academic literature. This is the first report of a case of FLA with disseminated intrahepatic foci.

**Table 1:** Reported cases of falciform ligament abscesses

Year	First author	Age, sex	Etiology	Treatment	Microbiology
1956	Hennessey [1]	Document unavailable			
1976	Charuzi [2]	74, F	Cholecystitis	FLR	Sterile
1980	Doscher [3]	79, M	Cholecystitis	FLR, cholecystectomy	*C. perfringens*, *B. fragilis, E. coli*
1981	Sones [4]	71, M	Cholecystitis	NR	NR
1988	Watson [5]	84, F	Unclear	FLR, cholecystectomy	Sterile
1988	Migliaccio [6]	NR, F	Infarction	FLR, cholecystectomy	NR
1989	Mori [7]	66, M	Hepatic abscess	Drainage	NR
1989	Mori [7]	59, F	Cholecystitis	Cholecystectomy	NR
1989	Mori [7]	66, F	Cholecystitis	Conservative	NR
1992	Brock [8]	96, F	Pancreatitis	FLR	*C. perfringens*, *S. epidermis*, *E. faecalis*, *Enterobacter Klugaria*
1996	Ko [9]	60, F	Bile stasis	FLR, left hepatectomy	*Bacteroides Streptococcus*
2002	Losanoff [10]	18, M	Unclear	FLR	*E. coli*
2003	de Melo [11]	65, M	Cholecystitis	Drainage, cholecystectomy	NR
2004	Martin [12]	52, F	Umbilical injury	FLR	NR
2008	Tsukuda [13]	70, F	Unclear	FLR, cholecystectomy	*S. epidermidis*
2009	Arakura [14]	63, M	Cholangitis	Conservative	S. anginosus*
2010	Czymek [15]	44, F	Unclear	FLR	*S. epidermidis*
2012	Warren [16]	73, M	Cholangitis	FLR, PD	Sterile
2015	Atif [17]	40, M	Pancreatitis	FLR	NR
2016	Sen [18]	40, M	Cholangitis	FLR, cholecystectomy	*E. coli*
2020	Fujikawa [19]	86, F	Idiopathic	Conservative	*S. marcescens*
2021	Bhattacharya [20]	69, F	NR	Conservative	NR
2021	Fang [21]	33, M	Cholecystitis	FLR, cholecystectomy	NR

*B. fragilis* *Bacteroides fragilis, C. perfringens Clostridium perfringens, E. coli Escherichia coli, E. faecalis Enterococcus faecalis,* *F* female, *FLR* falciform ligament resection, *M* male, *NR* not reported, *PD* pancreaticoduodenectomy, *S. anginosus Streptococcus anginosus, S. epidermidis Staphylococcus epidermidis, S. marcescens Serratia marcescens*

*Bile and bloodstream

The vascular and lymphatic networks that incorporate the falciform ligament connect it to the liver, diaphragm, retroperitoneum, mediastinum, abdominal wall, and chest wall. Its arterial blood flow is supplied by the left inferior phrenic artery, the middle hepatic artery, and, to a lesser extent, the left and right hepatic arteries [22–24]. These have anastomoses with terminal branches of the internal thoracic artery [25]. The veins of the falciform ligament are known as the vein of Sappey and vein of Burow. These drain into the left inferior phrenic vein and liver parenchyma (or peripheral portal veins), and communicate with the intrathoracic vein and epigastric vein [24–26]. The superficial lymphatics of the liver drain lymph along the falciform ligament and both down toward the abdominal wall and up to the parasternal lymph nodes [27]. Because of the presence of these vascular networks, FLA is believed to be caused by the transmission of bacteria from surrounding structures. In pediatric cases, omphalitis (infection of the umbilicus) is thought to be the causative primary disease that passes the infection to the falciform ligament, resulting in FLA [28]. However, many of the case reports of adult cases trace FLA back to primary hepatobiliary infections (Table 1). In the patient discussed herein, there were no obvious signs of cholecystitis or cholangitis through imaging studies. However, as *Pseudomonas aeruginosa* was detected both in the bile of the gallbladder and the abscess of the falciform ligament, bile infection likely triggered the development of the disease. Regarding the disseminated intrahepatic foci, continuous thickening of Glissonian tissue from the abscess to the disseminated foci in the liver was observed in the resected specimen, suggesting that bacteria traveled into the liver via vessels in the Glissonian tissue.

Although FLA is rare, diagnosis is relatively easy in cases with typical image findings as an abscess usually has a well-defined enhanced capsule wall and internal fluid and air content. In this case, small cystic lesions within the falciform ligament mass suggested the possibility of an abscess. Because antimicrobial agents were initially effective, we first diagnosed the mass as an infection of the falciform ligament. However, enlargement of the mass and a new nodule in the liver on CT images after initial treatment implied a malignant tumor. Tumors of the falciform ligament are uncommon; nonetheless, a metastatic case of intrahepatic cholangiocarcinoma in the falciform ligament with similar imaging findings to our case has been reported [29].

While conservative management with puncture or drainage is reported to be effective for FLA, many recommend surgical treatment. In our case, conservative treatment was only able to provide temporary relief from the disease and it soon recurred, leading to eventual surgical treatment. Conservative treatment for abscesses often requires prolonged antibiotic therapy. Relapse may occur during or after conservative treatment due to bacterial replacement or inadequate treatment. In our case, *Pseudomonas aeruginosa* was detected in the resected specimen and this is naturally resistant to the SBT/ABPC and CVA/AMPC used for effective initial treatment. Guidelines for the treatment of intra-abdominal abscesses emphasize the importance of source control [30]. The abscess in our case presented as a mass rather than capsulized fluid. Therefore, puncture or drainage would have been ineffective as methods of source control. Although multiple liver abscesses developed after surgery in our case, these quickly improved after the administration of antibiotics (levofloxacin). This demonstrates the importance of source control therapy for the treatment of FLA. As for the operative procedures, although simple resection of the falciform ligament, with or without cholecystectomy, depending on the presence of cholecystitis, has been performed in many reported cases, we performed resection with hepatectomy due to the possibility of cancer, based on the operative findings. In such instances, there should be an intraoperative pathological examination of frozen sections before determining the final procedure. If intraoperative pathological examination of the liver nodule had been performed first in the case presented here, left hepatectomy could have been avoided.

## Conclusions

We have here reported a case of FLA with a mass-like appearance and disseminated intrahepatic foci. It should be recognized that FLA can spread to both near and distant organs via its rich vascular and lymphatic networks.

## Data Availability

Data sharing does not apply to this article as no datasets were generated or analyzed in the current study.

## Abbreviations

CRP: C-reactive protein
CT: Computed tomography
CVA/AMPC: Clavulanic acid/amoxicillin
FLA: Falciform ligament abscess
MRI: Magnetic resonance imaging
SBT/ABPC: Sulbactam/ampicillin
